# Proceedings of the 2018 Asian American/Pacific Islander Nurses Association Conference: Local to Global—Future Directions for Research on Health Disparities: Editor’s Message

**DOI:** 10.31372/20180303.1004

**Published:** 2018

**Authors:** Jillian Inouye

The Asian American/Pacific Islander Nurses Association’s 15th Annual International Conference was held September 22–23, 2018 at the Hilton Garden Inn, located in Southpoint, Durham, S.C. with 43 research presenters. The conference theme was Local to Global: Future Directions for Research on Health Disparities with a dozen internationally well-known leaders around the world as keynote and special session speakers. Drs. Hyeoneui Kim, PhD, MPH, RN; Duke University School of Nursing and Jeeyae Choi, PhD, RN; University of North Carolina Wilmington School of Nursing were co-chairs and led the conference topics which addressed the current trends and future directions of health disparity research among AAPIs. The abstracts published here represent concurrent sessions focusing on the topics of: Nurses as Leaders- from Bedside to Board Room Moderator; Emerging Evidence and Future Directions of Clinical Practice; Paradigm Shift in Nursing Education to Influence Patient and Staff Safety and Patient Care Outcomes; Culturally Tailored Chronic Disease Care to Improve Patient Satisfaction and Patient Care Outcomes; The Use of Technology in Nursing Research; and Leveraging Technology for Practice, Research, Education. As the only journal focusing on (API) health and nursing we hope these topics give a flavor of areas important to API.

Jillian Inouye, PhD, APRN, FAAN

Editor

**Abstract 1**

Nursing Informatics skills for clinical nurses at the bedside: Developing and Implementing Nursing Informatics Competency Assessment Tool (NICAT)

**Alphonsa Rahman, DNP, APRN-CNS, CCRN^1^**

^1^Johns Hopkins Bayview Medical Center, Baltimore, MD, USA

**Background:** Informatics competency in clinical nursing is crucial to providing safe patient care, improving quality, and reducing healthcare costs. Assurance of informatics competency in a workforce with increasingly diverse educational preparations, demographics, and informatics skills poses major challenges. With no baseline competency assessment, developing an education and training program that meet individual needs and provide maximum benefits is difficult. An extensive literature search failed to find an existent nursing informatics assessment tool relevant to bedside nurses. The question addressed was the lack of nursing informatics competency assessment tools applicable to bedside nursing.

**Objective:** The objective of this study was to develop and review an evidence-based nursing informatics competency assessment tool (NICAT) that self-assesses nurses’ competency in computer literacy, informatics literacy, and informatics management skills applicable to patient care at the bedside based on the American Nurses Association’s *Standards and Scope of Practice and Technology Informatics Guiding Education Reform (2009)*.

**Methods:** The tool was developed by three rounds of review. Content validity was established by item analysis, relevancy scale, and validation by the identified experts from the organization’s Nursing Informatics Department (*n* = 4); the Department of Education, Practice, and Research (*n* = 8); the Clinical Outcomes Department (*n* = 1); and bedside nurses (*n* = 14). The project was guided by the Benner’s model and the Rosswurm and Larrabee framework.

**Result:** NICAT was designed and reviewed to address the individual educational needs of newly hired clinical nurses. This tool supports practices at the bedside by providing individualized education according to the results of a self-assessment.

**Implications for Nursing:** The NICAT will assist in preparing a workforce that is ready to deliver health care safely, efficiently, and cost-effectively by assessing newly hired nurse’s competency and guiding educators in developing future educational strategies based on individual needs. NICAT will assist in creating a workforce that is prepared to deliver patient care safely, efficiently, and cost-effectively in the increasingly technology-savvy environment of U.S. health care in the 21st century.

**Abstract 2**

Nurses as Community Leaders: Impacting Policies Where They Matter

**Emerson E. Ea, PhD, DNP, APRN, CNE^1^, Gloria Beriones, PhD, RN, NEA-BC, Jacqueline Michael, PhD, APRN, WHNP-BC**

^1^New York University, College of Nursing, New York, NY, USA

**Background:** Nurses have been key players in improving the lives of the communities where they live, work, play, and pray. Although there are many exemplars cited in the literature on how nurses impact their communities, there is a dearth of information that specifically highlight the contributions of nurses that address the health and well-being of the Asian American population, especially those Asian American subgroups that are underserved, underrepresented, and face challenges that relate to healthcare access and health inequities.

**Purpose:** This presentation aims to address that gap by showcasing exemplars of nurse-led community initiatives and their outcomes that have significantly contributed in enhancing the lives of Asian Americans. The presenters will also discuss the global reach and impact of these community-based initiatives.

**Results/Discussion:** Examples of community initiatives and their outcomes that will be presented include: (a) a multi-disciplinary collaboration that led to an increased availability of heart healthy options served during congregation meals at faith-based organizations and at select Filipino restaurants in New York City, (b) a culturally-tailored heart health education campaign aimed to improve heart health self-efficacy among Filipino Americans, (b) partnerships aimed at providing compassionate care and support to enhance the quality of life of Asian American patients diagnosed with cancer in Houston, Texas, and (c) programs that support the leadership skills and professional development of Filipino and Indian American registered nurses.

**Implications for Nursing:** Nurses who lead community-based initiatives in their roles as researchers, educators, clinicians, and/or policy influencers play key roles in addressing health inequities especially among Asian American subgroups that are underserved and underrepresented.

**Abstract 3**

Mentoring Nurses in Therapeutic Compassion as a Means to Address Moral Dejection: An Ethical Assessment Model that Positively Impacts Patient Safety and Satisfaction

**Geraldine Hider, RN, MS Disaster Medicine, MS Management^1^, Donald Hoepfer, MA Philosophy^2^**

^1^Mayo Clinic, MN, USA

^2^Carroll Community College, Westminster, MD, USA

**Introduction:** The authors offer a model for performing an ethical assessment that relies on compassion along with utilitarian and deontological analyses. Though compassion is thought by some to be fatiguing, the authors’ research has shown compassion to be a therapeutic skill with important implications for nurses. This presentation offers a unique perspective drawn from the clinical setting and informed by academic philosophy in order to present a nuanced awareness of compassion as a therapeutic skill that must be distinguished from empathy, which can be a catalyst for moral dejection.

**Description of basic concepts:***Empathy* is an experience that follows the observation of pain in another. This experience is characterized by a feeling of pain on the part of the observer that mirrors the pain felt by the other person. *Compassion* is the willingness to be responsive to another person who suffers and to establish an interpersonal connection that affirms the importance and worth of that person. *Moral dejection* is a general term referring to a feeling of low spirits extending from a realization that one cannot bring about the moral good that one wishes.

**Implications for Nursing:** Educational interventions to develop skills in therapeutic compassion will develop understanding of the nature of compassion and distinguish it from empathy. Feelings of moral dejection can arise from moral reactions that can be addressed through ethical assessment. All too often, moral reactions are confused for ethical perceptions. Nurses who employ the Ethical Assessment Model will develop improved skills in communication as well as skills that contribute to moral satisfaction and counter burnout, stress, and vertical and horizontal violence. The presentation offers interventional guidelines in the form of conceptual models that can be utilized in mentoring nurses. Compassion is situational and spontaneous. Though it can’t be given as a protocol, it can be cultivated, mentored, and used therapeutically. The aim of the models are to encourage improved understanding, collegial interaction and support, and, ultimately, to encourage a culture of compassion. Progress toward such a culture is evidenced by improved morale and by improved metrics regarding patient safety, and patient and family satisfaction.

**Abstract 4**

Feasibility of developing iDEStress (individualized Detection and Evaluation of Stress), a mobile stress management application

**Hyeoneui Kim, RN, MPH, PhD^1^, Ted Ochiai^2^, Victoria Ngo^3^, Beverly Morris, RN, CNP^4^**

^1^School of Nursing, Duke University, Durham, NC, USA

^2^Marketo, Inc., San Mateo, CA, USA

^3^School of Nursing, University of California Davis, Davis, CA, USA

^4^Veterans Affairs Hospital, San Diego, CA, USA

**Objectives:** The purpose of this study was to test the feasibility of developing a mobile application that offers personalized monitoring and mitigation of stress for nurses at work. This early work focused specifically on developing stress algorithms, strategies for secure data management, and an android application that demonstrates real time transmission of data and display of calculated stress level. The Joint Commission recognized the workplace fatigue and burnout as an important patient safety concern. The American Nurses Association also called for actions to improve wellbeing of nurses in the workplace through the “Healthy Nurse, Healthy Nation” initiative in 2012 to mitigate unhealthy coping with work related stress and burnout among nurses. Policies and regulations that prevent overwhelming working hours and schedules are in place to address these issues. However, few attempts have been made to help nurses actively manage their workplace stress at the personal level.

**Methods:** After finalizing the functional requirements of iDEStress using the storyboarding method, we built a prototype iDEStress application connected to *Microsoft Band 2*. We developed the algorithm that aggregates the sensor data into stress levels and tested it with 200,000+ data points captured from three volunteers using *Microsoft Band 2*. This stress algorithm was implemented in a secure local data server. We also wrote an algorithm to transmit the sensor data sent to a smartphone from *Microsoft Band 2* to the local data server every 2 minutes.

**Results:** The prototype iDEStress application successfully transmitted the sensor data to the local data server as scheduled and displayed the calculated stress levels back in real-time. The stress algorithm showed only a moderate level of correlation with the actual perceived stress level of the volunteer subjects, indicating more extensive testing or revamping of the algorithms is needed. Ensuring secure data transmission is another area that requires further investigation.

**Conclusion:** Developing the iDEStress application is potentially feasible. Success of iDEStress will add a new angle to approaching the challenges in nurses’ workplace wellbeing and patient safety associated with nursing burnout.

**Implications for Nursing:** Studies showed that nurses’ stress and burnout are associated with increased medication errors and workplace injuries. A mobile sensor based personalized stress monitoring and management system can help improve nurses’ workplace wellbeing.

**Abstract 5**

Long-Term Impact of Mild Traumatic Brain Injuries on Cognitive, Psychosocial, Balance Performance and Epigenetics

**Hyunhwa Lee, PhD, MSN, APRN, PMHNP-BC^1^, Sungchul Lee, PhD^2^, Laura Salado^3^, Jonica Estrada^4^, Alex Santos, PhD^5^, Katrina Isla, BSN^1^, Joseph Lao, OD^6^, Sambit Mohapatra, PT, PhD^7^, Charles Bernick, MD, MPH^8^**

^1^School of Nursing, University of Nevada, Las Vegas (UNLV), USA

^2^Department of Computer Science, University of Wisconsin Whitewater, USA

^3^Florida International University, USA

^4^School of Life Sciences, UNLV, USA

^5^College of Health and Human Services, Western Michigan University, USA

^6^Optic Gallery on Stephanie, USA

^7^Department of Rehabilitation & Movement Science, University of Vermont, USA

^8^Cleveland Clinic Lou Ruvo Center for Brain Health, USA

**Objectives:** Over 80% of the 1.7 million traumatic brain injuries (TBIs) occurring annually in the U.S. are considered ‘mild’ (mTBI) or concussion. The increasing incidence and growing health impact of TBI may result from insufficient information regarding how to precisely diagnose and assess TBI, especially mild to moderate, which has a heterogeneous symptom profile and disease trajectory. This study compared and identified any deficits in cognitive, psychosocial, visual functions, and balance performance between mTBI and controls. Global DNA methylation ratio in blood was also compared.

**Methods:** Participants were recruited from a local university (UNLV) for a translational clinical research protocol for mTBI where cognitive (measured by NIH toolbox), psychological (measured by the Patient Reported Outcomes Measurement Information System), visual function (measured by King-Devick Test), postural balance performance (measured on a force plate), and epigenetic blood marker (global DNA methylation) were compared between mTBI and controls.

**Results:** Twenty-five volunteers participated (*M* = 25.0 years of age; *SD* = 5.96) and all provided informed consents based on procedures approved by the UNLV Institutional Review Board (protocol #1048342). mTBI cases consisted of 11 individuals (72.7% males; *M* = 28.7 years of age; *SD* = 5.92) with a self-reported history of single or multiple injuries (63.6% multiple; 2.3±1.25 injuries), including sports-related activities and military operations. The average time elapsed since the last injury was 7.1 (*SD* = 6.77) years. Controls consisted of 14 individuals (35.7% males; *M* = 22.0 years of age; *SD* = 4.13). Only age was statistically different between the 2 groups (*p* = .003) among all demographic variables. mTBI cases reported significantly poorer episodic memory (*p* = .022), severer anxiety (*p* = .022) and more sleep disturbance problems (*p* = .023), and higher blood global methylation level (*p* = .030). No significant differences were found in visual function and postural balance; however, there was a trend that mTBI individuals showed a decrease in the mean frequency containing 50% of the power spectrum density in medio-lateral direction during bipedal stance (*p* = .079).

**Conclusion:** These findings validate changes in cognitive, psychosocial, and global DNA methylation long after acute mTBI.

**Implications for Nursing:** Findings from this study could contribute to early detection of symptoms for ensuring early clinical intervention and rehabilitation.

**Abstract 6**

Biobehavioral Correlates: Women in Low Socioeconomic Class with Chronic Low Back Pain

**Jennifer Kawi, PhD, MSN, APRN, FNP-BC^1^, Nada Lukkahatai, PhD, MSN, RN^2^, Hyunhwa Lee, Ph.D., MSN, APRN, PMHNP-BC^1^**

^1^University of Nevada, Las Vegas, School of Nursing

^2^Johns Hopkins University, School of Nursing

**Purpose:** Health care disparities abound in pain management. Minimal studies have looked at chronic low back pain (CLBP) among women in low socioeconomic class particularly involving extensive assessment of various biobehavioral factors. This study focused on evaluating various biobehavioral correlates influencing women with CLBP belonging to low socioeconomic class. These include evaluation of global DNA methylation levels, one of the key measures of alterations of the epigenome; modifications influence the functions of genes that can consequently impact pain and treatment response.

**Methods:** This preliminary, descriptive, and cross-sectional study that was IRB-approved and conducted in a comprehensive pain center. The National Institute of Nursing Research Symptom Science Model guided this study. Participants with CLBP in low socioeconomic class completed questionnaires on biobehavioral factors (*N* = 39). Peripheral blood was drawn to evaluate global DNA methylation using Enzyme-linked Immunosorbent Assay. Data were analyzed using SPSS 22.

**Results:** Participant mean age was 50; 92.3% were non-Hispanics, 38% were Whites, majority lived below poverty level, attained high school or less, and unemployed. Mean pain intensity was 7.8 and CLBP duration was 11.7 years. Other mean findings were: BMI = 32.2, opioid use = 51.3 morphine milligram equivalents/day, time spent in aerobic exercise = 96.54 minutes/week, self-efficacy = 4.1 (maximum of 10), pain catastrophizing = 29.5 (maximum of 54), and global methylation = .47. Quantified global methylation levels significantly correlated with exercise (*r* = .399, *p* = .012) and this correlation was moderated by race. Greater exercise among Whites was associated with higher levels of global methylation.

**Conclusion:** The key long-term objective was to apply findings toward precision medicine in global pain health. Findings can assist in identifying high risk groups and targeting specific interventions toward better pain management particularly in addressing pain care disparities and documented under treatment among women with low socioeconomic status.

**Implications for Nursing:** Modifiable risk factors such as high opioid use, BMI, and less exercise are essential targets for nurses and other healthcare providers, with consideration for the influence of race. These preliminary results indicate the need for larger, longitudinal, and interventional studies to facilitate better clarity on the role of epigenomics among women with CLBP.

**Abstract 7**

Effect of Comorbid Depression in Diabetes Care Outcomes

**Letha M. Joseph, MSN, DNP, APRN, AGPCNP-BC^1^, Diane Berry, PhD, ANP-BC, FAANP, FAAN**

^1^Durham Veterans Affairs Medical Center, Durham, NC, USA

**Introduction:** Diabetes is a highly prevalent chronic disease. According to Chronic Care Model (CCM), better chronic disease outcomes are achieved when productive interactions take place between a prepared and proactive practice team and empowered and prepared patients. The presence of diabetes almost doubles the odds of developing depression. Comorbid depression negatively affects diabetes self-management and leads to poor diabetes care outcomes and a higher utilization of healthcare services.

**Description of Concept:** According to Social Signal Transduction Theory of Depression, social stressors such as social rejection, isolation, exclusion and social adversity up-regulate the immune system which leads to the production of pro-inflammatory cytokines. These cytokines signal the brain to induce behavioral and emotional changes leading to depression. These mechanisms explain how social stressors and chronic inflammation in patients with diabetes increase their vulnerability to comorbid depression.

Unidentified depression exists in patients with diabetes. Depressive symptoms in adults with diabetes negatively affect their self-management and adherence to diabetes treatment, which leads to poor glycemic control, and microvascular and macrovascular complications. According to the REasons for Geographic And Racial Differences in Stroke (REGARDS) study, comorbid stress and depression are common among patients with diabetes and are associated with poor cardiovascular outcomes. Patients with co-morbid depression tend to report poor Health Related Quality of Life and suffer from more diabetes related symptoms and utilize health care services more frequently than patients with diabetes who do not have any depressive symptoms. Even minor depression could affect diabetes self-management. However, studies reported the presence of undiagnosed depression in nearly 45% of patients with diabetes.

**Practice and Implications for Nursing:** Routine screening and management of depressive symptoms may improve diabetes care outcomes. There are various factors related to providers and patients which are potential barriers in integrating depression management in diabetes care. System level concerns such as time constraints, competing clinical expectations, general practitioners’ reluctance in initiating depression care and difficulty in collaborating with mental health care are potential barriers. The indication of depression management is strong enough to overcome the system barriers. Nurses and advanced practice nurses have an important role in recognizing and managing depressive symptoms in these patients.

**Abstract 8**

Impact of Health Insurance on Colorectal Cancer Screening and Healthcare Service Utilization among Korean Immigrants: A Mixed Method Approach

**Moonju Lee, PhD, RN^1^, Carrie Jo Braden, PhD, RN, FAAN^1^**

^1^UT Health San Antonio School of Nursing

**Purposes:** Lack of healthcare access is considered one of most common barriers in preventive care, such as colorectal cancer (CRC) screening, among Korean Immigrants (KIs). However, the relationship between access and healthcare services utilization is still unclear and inconsistent among KIs. To investigate the impact of health insurance on CRC screening and to explore healthcare service utilization experiences among KIs.

**Methods:** The Social Determinants of Health model provided the conceptual framework. A survey of 498 KIs aged 50 and older was conducted along with 18 individual interviews. For data analysis, chi-square and multiple regression for quantitative data and content analysis for qualitative data were used.

**Results:** The study sample reported using the following CRC screening method: fecal occult blood test (37.6%), colonoscopy (62.4%) and sigmoidoscopy (6%). Eighty-five percent reported having health insurance, major insurance was Medicare (45.4%), purchased a health plan (12%), Medicaid (11.6%), employers (11.4%), Affordable Care Act (10.4%), and military health care (6.4%). The interview study identified barriers and facilitators for CRC screenings and healthcare services utilization. Barriers were reported as 1) being unfamiliar with healthcare systems, 2) lack of health insurance, 3) lack of knowledge of insurance coverage and preventive care guidelines, 4) language and communication barriers, 5) perceptions of preventive care, and 6) lack of healthcare provider’s recommendation for CRC screenings and preventive care. Facilitators were reported as community education programs, free health services, Korean newspapers, and family and friends’ experiences. About 83% of those who had undergone CRC screening had it performed in South Korea and 94.4% of them had never received a physician’s recommendation for CRC screening in the U. S.

**Conclusion:** The rates of CRC screening are significantly lower among KIs than the general U.S. population and the national goals of “80% by 2018”. Although the insured rates had significantly increased with a contribution of Affordable Care Act, KIs still face health disparities in CRC screening and healthcare service utilization. Most KIs do not effectively utilize the health insurance to manage their health. It is necessary to develop the culturally and linguistically tailored interventions to reduce cancer health disparities and improve health literacy for healthcare service utilization.

**Implications for Nursing:** This study enhances knowledge of unique health beliefs and behaviors related to CRC screening and health services utilization of KIs to nurse clinicians and researchers.

**Abstract 9**

Integration of Interprofessional Education in Health Sciences Curriculum: Addressing Health Disparities

**Rowena T. Bermundo, MN, RN^1^**

^1^American Sentinel University, Aurora, CO, USA

**Introduction:** The Institute of Medicine recommends interprofessional education (IPE) as a collaborative approach to cultivate healthcare students as future interprofessional team members. Increasing globalization of healthcare and the diversity of this nation’s population mandates attention to provide safe, high-quality care. The professional nurse practicing in a multicultural environment must possess the skills to provide culturally sensitive and appropriate patient care. Cultural competency is a significant component of patient-centered care in a complex and diverse healthcare setting.

A partnership between Colleges of Nursing and Pharmacy at Roseman University of Health Sciences was formed with the primary goal to provide opportunities for students to work on patient case studies that deals with health disparity issues. From the case studies, the students address the healthcare needs of patients from different races, ethnicity, religion, sexual orientation, and disabilities including communication barriers. The students from both colleges worked together to offer culturally proficient strategies and presented together in class. Due to the success of this type of learning approach, the Colleges of Medicine and Dentistry will also be participating in this IPE initiative in the near future.

**IPE Concept:** Interprofessional education is the involvement of educators and learners from two or more health professions and foundational disciplines to jointly create and foster a collaborative learning environment. Cultural competency is the ability of the healthcare provider to efficiently deliver care to patients with diverse values, beliefs, practices, and behaviors including social, cultural, and linguistic needs.

**Implications for Nursing:** In IPE, nursing students will learn to value teamwork and how to work with other professionals. It is crucial for nurses to focus more on valuing the patients than just determining the correct treatments. The addition of collaboration among healthcare professionals can lead to develop culturally appropriate healthcare system and workforce that deliver the highest quality of care to every patient.

**Abstract 10**

Servant Leadership in Nursing

**Ruth W. Mustard, RN, MSN NEA-BC^1^**

^1^Dorn Veterans Affairs Medical Center, Columbus, SC, USA

**What is Servant Leadership?** The concept of servant leadership began with Robert Greenleaf in the 1970’s. A seasoned nursing leader in the Veterans Health Administration (VHA) for 38 years describes the journey of servant leadership. The focus of the presentation is on servant leadership at the nurse manager level; however, anyone can be a servant leader. Servant leadership is not subservience, nor are nurses reverting to a role of that of handmaiden to the physician. Quite the contrary, servant leaders are those who are leading teams that have the characteristics of listening, empathy, healing, awareness, persuasion, conceptualization, foresight, stewardship, commitment to the growth of people, and ability to build community.

**Servant Leadership in the Veterans Health Administration:** The VHA has utilized a score on the annual all employee service called the servant leader index that is comprised of ten items in two content areas of supervisor and workgroup perceptions. The items combine to form an overall score of 0 (worst) to 100 (best), and include fairness, advocacy, favoritism, psychological safety (disagreement and comfort talking), employee development, performance goals, conflict resolution, workgroup communication, and accountability. This score is an indication of how well managers accomplish organizational goals through the empowerment of others. Staff perceptions of managers with higher servant leadership orientation demonstrates significant positive impact on employee job satisfaction.

**The Nurse Manager as a Servant Leader, Implications for Nursing:** The Nurse Manager heavily impacts job satisfaction, and VHA has determined that a higher servant leadership score is desirable and significantly contributes to achievement of job satisfaction and organizational goals. Interventions are needed to improve satisfaction and servant leadership characteristics. Care of the staff through managers who are committed to serving them and meeting their emotional needs may be the competitive advantage in health care in this century.

**Abstract 11**

The Use of mHealth to Assist Self-Management and Access Services in a Rural Community

**Reimund Serafica, Ph.D., MSN, RN^1^, Jillian Inouye, Ph.D., MSN, FAAN, Nada Lukkahatai, Ph.D., MSN, RN, Nafanua Braginsky, Ph.D., DNP, APRN, Misty Pacheco, DrPH, MHA, Katharyn F. Daub, EdD, MNed, CTN-A, RN**

^1^University of Nevada Las Vegas, School of Nursing, Las Vegas, NV, USA

**Purpose:** The purpose of this study was to explore the needs, barriers and facilitators of using mobile health (mHealth) technology to assist low income Asian Pacific Islander (AAPI) participants living in rural Hawai‘i. Our specific aims were (1) to conduct focus groups and eHealth assessments in a low-income rural setting on Hawai‘i Island (the Big Island); (2) to identify the unique barriers to and facilitators of shared medical decision making, adherence, and effective disease management among the patients.

**Methods:** Three focus groups consisting of patients, family support/significant others and providers (*N* = 19) was conducted to access the unique needs of low income AAPI patients. The responses of all group members were compared and contrasted across the interviews. During the participant checking, the participants reviewed the themes generated and agreed on their faithfulness to their own experiences. The explicitness of the research data was achieved by maintaining an audit trail of researcher-generated data, including observational field notes. The eHealth literacy scale was also measured among participants in the patients and family support / significant other group.

**Results:** The majority of participants in the patients group were Asian and Native Hawaiian or other Pacific Islander (71%), while 50% of participants in the health care provider session were Hispanic and White. The educational level of the participants in the patient and family supporters/significant others groups ranged from some high school to some college. The total eHealth literacy means were 23.57 (SD = 9.71) among participants in the patient group and 34.50 (SD = 7.78) in the family support/significant others group. The qualitative analysis yielded categories with three main themes: Value of mHealth, Stumbling Blocks to mHealth, and mHealth Wish List. The participants also highlighted the costs, privacy and security concerns and limited data and Wi-Fi coverage.

**Finding/Conclusions:** While the impact of the technology on patient outcomes is inconsistent, most participants seem willing and eager to learn and use mHealth for education and communication purposes. Patients and their support believed that it is beneficial in understanding their overall health status and disease management.

**Implications for Nursing:** Practice implications include uses of these findings to integrate future versions of mHealth that will promote effective communication and information specifically to the diverse vulnerable population.

**Abstract 12**

Informatics Support for Diabetic Retinopathy Screening

**Ricky K. Taira, Ph.D.^1^, Omolola Ogunyemi, Ph.D.^1,2^, Lauren P. Daskivich, MD^3^, Alex Bui, Ph.D.^1^**

^1^Department of Radiological Sciences, University of California Los Angeles, Los Angeles, CA, USA

^2^Center for Biomedical Informatics, Charles Drew University of Medicine and Science, Los Angeles, CA, USA

^3^Los Angeles County Department of Health Services, Los Angeles, CA, USA

**Purpose:** To investigate a system-wide approach to identify patients with early signs of diabetic retinopathy based on digital retinal screening examinations.

**Methods:** We are in the process of designing and implementing an end-to-end information management system with the goal of early detection of diabetic retinopathy. The design requirements include assumptions that patient eye examinations can originate in various safety-net clinics where an ophthalmologist or other eye specialist is not immediately available and which may delay access to time-sensitive treatments. The core information system is based on the non-profit foundation software called EyePACS. We are currently working with computer vision experts to automatically interpret digital retinal color fundus images according to their probability of showing a diagnosis of: a) no disease; b) mild non-proliferative retinopathy; c) severe non-proliferative retinopathy; d) proliferative retinopathy.

**Results:** An important milestone of this project is the commitment to a team approach toward clinical implementation. Team members include a number of clinical ophthalmologist, clinical/research coordinators, the director of the Los Angeles County Eye Health Program, medical informaticians, and computer scientists. We are working on the important fronts of clinical concerns and requirements, informatics issues (e.g., EyePACS, ontologies, structured reporting, information interfaces), technical issues (e.g., deep learning image analysis methods) and research issues (e.g., disease modeling), all toward the goal to improve the availability of high quality screening for health care environments that may be lacking in resources. A preliminary evaluation to determine whether a primary-care based teleretinal screening program can improve timeliness to care was recently conducted showing a 16% increase in the rate of screening and a reduction of 89% in wait time.

**Conclusion:** Architecting a teleretinal screening program must include an end-to-end solution based on multi-faceted requirements that includes clinical, sociological, technical, and economic considerations.

**Implications for Nursing:** We believe that diabetic patients would ultimately benefit from having a low cost, minimally intrusive process that can be widely implemented in diverse environments, including nurse managed health centers. Such a system could improve screening rates and provide mechanisms for efficient triaging to speed up diagnosis and management.

**Abstract 13**

What Should We Know to Culturally Tailor Technology-Based Interventions to Racial/Ethnic Minorities?: A Case of Cancer Pain Management Program for Asian American Breast Cancer Survivors

**Sangmi Kim, PhD, MPH^1^, Xiaopeng Ji, PhD, RN^2^, Eunice Chee, BSE^3^, Ting Bao, DABMA, MS^4^, Jun J. Mao, MD, MSCE^4^, Wonshik Chee, PhD^1^, Eun-Ok Im, PhD, MPH, RN, CNS, FAAN^1^**

^1^Duke University School of Nursing, Durham, NC, USA

^2^University of Delaware School of Nursing, Newark, DE, USA

^3^North Carolina State University School of Engineering, Raleigh, NC, USA

^4^Memorial Sloan-Kettering Cancer Center, New York City, NY, USA

**Background:** Technology-based interventions are accepted as preferred and effective means in racial/ethnic minority communities to enhance their various health outcomes through information and coaching/support. Little discussion, however, exists on practical challenges encountered over the course of such interventions beyond ethical issues or recruitment and retention of study subjects.

**Objectives:** This presentation aims to discuss practical issues identified during a pilot study on a technology-based cancer pain management program for Asian American (Chinese, Korean, and Japanese) breast cancer survivors and to provide useful advice for future technology-based interventions among racial/ethnic minorities.

**Methods:** The research diaries written by individual research team members and the meeting logs were reviewed and analyzed by using Weber's content analysis. In the analysis, each word was used as a unit to generate codes, which were then categorized into themes, reflective of practical issues in technology-based interventions.

**Results and Discussions:** Six major problems were: (1) heterogeneous sub-cultures (e.g., language, network pattern, etc.) among Asian American breast cancer survivors; (2) survivors’ changing interests and health needs along the course of treatment and recovery; (3) limited accessibility to culturally congruent and reliable Internet resources from survivors’ countries of origin; (4) difficulty building trust with study participants and gatekeepers; (5) varying degree of adherence, lower retention rate, and authenticity issues; and (6) culturally sensitive usages of words. Future technology-based interventions for racial/ethnic minorities consider: (a) reflecting the diversities among and within the sub-ethnic groups of participants in the intervention's design and implementation; (b) catering to each participant's needs by considering one's unique treatment and healing process; (c) providing accurate and reliable Internet resources from participants’ countries of origin through careful review of the content by bilingual researchers with subject-matter expertise; (d) using culturally matched research team members to gain trust from potential participants and gatekeepers; (e) developing a protocol to prevent and deal with fake identity cases; and (f) improving cultural competency of the intervention through collaborating with researchers with cultures and languages of the target populations.

**Conclusions:** Future technology-based programs for racial/ethnic minorities need to consider these challenges and subsequent suggestions for design and implementation.

**Abstract 14**

Meaningful Recognition: Is the essential nature of this Healthy Work Environment component fully understood by staff nurses and leaders?

**Usha Koshy Cherian, DNP, RN, CNL, CCRN, NEA-BC^1^**

^1^Wm JB Dorn Veterans Affairs Medical Center, Columbia, South Carolina, USA

**Purpose:** According to AACN (2005), Meaningful Recognition (MR) for job performance, is an essential element of a Healthy Work Environment (HWE) and is central to nurses’ satisfaction and retention, patient satisfaction and outcomes, and organizational financial viability. However, even after a decade of implementing HWE initiative, there is little evidence to guide clinical practices related to MR strategies. Hence recognition is given based on assumptions, traditions, and previous experiences, which may or may not be meaningful to their nursing staff members. The purpose of this project was to explore the perception of MR among staff nurses and nurse leaders and identify innovative methods for recognizing nurse’s contributions in ways that are valued by the individual.

**Method:** The project utilized a mixed method approach among a convenience sample of nurse leaders and staff nurses in the Intensive Care Units (ICU) of an academic medical center. Twenty-six nurses participated in seven focus group interview (FGI) sessions, grouped by positions. Ninety-five nurses participated in the HWE and Recognition surveys administered via Qualtrics software.

**Theoretical Framework:** Theory of Motivation by Maslow (1943) and Schein’s Organizational Culture Theory provided the framework for this project.

**Results:** Thematic analysis of FGI discussion yielded eight themes. The results of the Recognition survey was similar to the FGI theme ‘ways to give MR’, which confirmed that salary commensurate to performance scheduling flexibility, opportunities for growth, private verbal feedback and written and public recognition were the most meaningful methods of recognition.

**Conclusions:** HWE cannot be achieved when MR is considered optional. Nursing leadership should focus on developing strategies to provide MR in a consistent and systemic manner.

**Implications for Nursing:** Nurses need to be trained in finding intrinsic form of MR and reflective practices as a way of MR. Ways to provide MR should be added as an essential competency for nurse leaders, so as to improve the satisfaction, engagement, productivity and retention among nurses.

**Abstract 15**

A Technology-Based Cancer Pain Management Program: Preliminary Effectiveness in Asian American Breast Cancer Survivors

**Wonshik Chee, PhD^1^, Yaelim Lee, PhD, RN^2^, Xiaopeng Ji, PhD, RN^3^, Eunice Chee, BSE^4^, Eun-Ok Im, PhD, MPH, RN, CNS, FAAN^1^**

^1^School of Nursing, Duke University, Durham, NC, USA

^2^Chung-Ang University, Seoul, Republic of Korea

^3^Univ. of Delaware, Newark, DE, USA

^4^North Carolina State University, Raleigh, NC, USA

**Significance:** With advances in computer and mobile technologies, the effectiveness of technology-based interventions has been supported in improving various symptoms including cancer pain and in changing health behaviors. With few technology-based programs for cancer pain management, the necessity of culturally tailored technology-based programs for cancer pain management of racial/ethnic minorities has been claimed.

**Objectives:** The purpose of this study was to determine the preliminary effectiveness of a technology-based cancer pain management program (CAPAA) on cancer pain experience of Asian American breast cancer survivors.

**Methods:** This was a pilot study using a randomized repeated measures pretest/posttest control group design among 94 Asian American breast cancer survivors. The instruments were: the Brief Pain Inventory-Short Form, the Support Care Needs Survey-34 Short Form, and the Mishel Uncertainty in Illness Scale-Community. The data analysis was conducted using descriptive and inferential statistics including repeated measures ANOVA.

**Results and Discussions:** Although no significant differences were found in cancer pain, there existed significant differences in perceived isolation (*F* = 9.937, *p* < 0.01), personal resources (*F* = 6.612, *p* < 0.05), support care need (*F* = 8.299, *p* < 0.01), and degrees of uncertainty (*F* = 8.722, *p* < 0.01) of the intervention group from *pre-test* to *post-test*. The perceived isolation scores changed more in intervention group (*M* = −0.43, *SD* = 0.79) compared with the control group (*M* = 0.11, *SD* = 0.20; *F* = 5.471, *p* < 0.05). The personal resource scores increased in the intervention group (*M* = 0.53, *SD* = 0.59) while they decreased in the control group (*M* = −0.11, *SD* = 0.68; *F* = 10.027, *p* < 0.01). The degree of uncertainty scores decreased in the intervention group (*M* = −0.49, *SD* = 0.87) while it increased in the control group (*M* = 0.05, *SD* = 0.37; *F* = 4.455, *p* < 0.05).

**Conclusions:** The findings supported the preliminary effectiveness of CAPAA on cancer pain experience of Asian American breast cancer survivors. It is necessary to test the CAPAA with diverse groups of Asian American breast cancer survivors through future studies.

**Abstract 16**

Association of Balance with Bone Mass, Lean Mass and Fat Mass

**Young-Shin Lee, PhD^1^**

^1^School of Nursing, San Diego State University, San Diego, CA, USA

**Background:** With aging, body composition changes to an unstable balance, which causes incidents and injuries among older adults; this is leading to serious, frequent medical and public health problems. Although maintaining balance is a key to prevent falls, there is no clear understanding between balance and body composition.

**Purpose:** The purpose of the study includes identifying the factors of balance performance and its associations with soft tissue components among Korean American women with three age groups.

**Design:** A cross-sectional descriptive study was conducted with 63 Korean American women divided into three age groups: 25-35, 45-55, and 65+ years old.

**Methods:** The following variables were collected: Fat mass and lean mass on the Bone mineral density screening sites, static and dynamic balance and physical performance tests.

**Results:** With increased aging, lean mass, fat and BMI were changed; and balance and physical performance decreased significantly. Dynamic balance and sitting on floor to standing were associated with lean mass and fat mass in total and appendicular sites, respectively.

In regression models for balance, younger age was the strongest predictor for static balance; greater appendicular LM and lesser percentage of fat on android for dynamic balance; and younger age and fewer percentage of fat on android were significant variables to explain better floor sitting to standing performance.

**Conclusions:** Only fat on android area was negatively associated with dynamic balance while other soft tissues did not reflect on static balance performance. Maintaining muscle mass and preventing fat accumulation during menopausal process is critical for stable balance.

**Implications for Nursing:** Education for strategies how to prevent fat accumulation in the android area is necessary to maintain balance and prevent falls.

**Abstract 17**

Professional Socialization among Intensive Care Unit Nurses in South Korea: Structural Equation Modeling

**You Lee Yang, PhD, RN^1^**

^1^Duke University School of Nursing, Durham, NC, USA

**Purpose or Objective:** The turnover rate of mid-level ICU nurses is increasing annually in South Korea. The absence of mid-level nurses leads to organizational and economic losses. Therefore, in order to manage ICU nursing staff effectively, it is necessary not only to secure competent nursing staff but also to ensure continuous and dynamic professional socialization. This study was to construct and test a structural equation model of professional socialization among clinical nurses in intensive care unit (ICU) based on Benner’s stages of nursing proficiency model.

**Methods:** A hypothetical model of professional socialization with associating factors (nursing competence, social recognition of nurse, nursing work environment, and job satisfaction) was developed. By quota sampling method, 232 ICU nurses across 22 tertiary hospitals were enrolled. The data collection was conducted from May to September, 2017, using a mail survey. The data were analyzed using structural equation modeling with the SPSS WIN 23.0 and AMOS 18.0 software program.

**Results:** The mean age of study participants was 28.9 (±5.10) years, 82.3% of participants had 4-year bachelor’s degree, and the mean years of ICU clinical experience was 3.4 (±3.72) years. In the theoretical model, nursing competence (*β* = .584, *p* = .003) and social recognition of nurse (*β* = .270, *p* = .003) had direct effects on professional socialization for 49.8%. Nursing work environment had indirect effects on nurses’ professional socialization (*β* = .152, *p* = .005). The pathway from professional socialization to relating factors were significantly different by nursing expertise (novice versus expertise groups).

**Conclusions:** This study results indicate the necessity of developing intervention to enhance the nursing competence appropriate for each level of nurses, and increase recognition or pride of the nursing job for successful professional socialization of ICU nurses.

**Implications for Nursing:** Based on the theoretical model of this study, systematic development of program to facilitate ICU nurses’ professions, competence, and socialization are expected in clinical and academic fields.

**Abstract 18**

Atrial Fibrillation and Adherence to Antithrombotic Guidelines in Home Based Primary Care Patients at Durham Veterans Affairs Medical Centre

**Achamma Abraham, MSN, FNP-C, WCC^1^, Radhika Kothappalli, PharmD, BCACP^1^, Jeanette Stein, MD^1^**

^1^Durham VA Medical Center, Durham, NC

**Purpose:** To determine if the Veteran patients with Atrial Fibrillation (AFib) receiving HBPC care are on guideline appropriate antithrombotic therapy. To identify the reason for non-adherence to guideline appropriate antithrombotic therapy in this population.

**Methods:** Researchers reviewed electronic health records of two hundred veterans enrolled in HBPC during 1/2017-6/2017. Data collected includes the diagnosis of Afib/ A flutter as documented in patient problem list and the status of oral anticoagulation prescription.

Researchers also used the tool CHADS2VASC to calculate thromboembolic risk. Veterans who scored ≥ 2 oral anticoagulants are recommended.

**Results:** A total of 39 veterans had an identified diagnosis of Afib in their electronic health records, 32 of which were on guideline appropriate treatment regimen, and seven were not on anticoagulant treatment due to reasons mentioned in their records.

**Conclusions:**

- One-fifth (20%) of chronically ill veterans on the Durham VA Medical Centre HBPC patients have Atrial Fibrillation.
- 82% of those HBPC Veterans are treated as per 2014 AHA/ACC/HRS guidelines with Warfarin and dose monitoring of INR/labs/medication adjustment.
- Guideline adherent antithrombotic treatment reduces stroke-risk and improves the outcome in patients with Afib.
- 18% of HBPC chronically ill veterans studied are not receiving guideline suggested antithrombotic treatment due to prior history of significant Gastrointestinal bleeding, dementia, not aligned with goals of care, and family preference for not having treatment with blood thinners.

**Implications for Nursing:** Several patients with Afib/mechanical heart valve are on Warfarin, the oral anticoagulant. It is an important point to consider when prescribing medications for this vulnerable population since there are several medications including antimicrobials which increases their risk for bleeding. Frequent patient and family education on diet modification and signs of occult bleeding is important. It is important to monitor INR and haemoglobin periodically to recognize potential complications in a timely manner.

**Abstract 19**

Risk Perception of Developing Diabetes in Vietnamese Americans with Prediabetes

**Angelina Nguyen, MSN, RN, CNE^1^**

^1^Arizona College, Las Vegas, NV, USA

**Background:** Diabetes is a potentially crippling, chronic disease that may be prevented through health-promoting behaviors. There are 86 million American people living with prediabetes, and nearly one-third will develop diabetes within five years without lifestyle changes. Vietnamese Americans (VnA) are a vulnerable population with higher diabetes prevalence rates despite lower mean body mass index compared to Non-Hispanic Whites.

**Purpose:** The purpose of this literature review was to explore the risk perception for developing diabetes in VnA with prediabetes. Risk perceptions are the general and personal thoughts and feelings that may include worry, anxiety, or even optimism about the susceptibility, vulnerability, and likelihood of developing a disease and the disease’s severity that are both instinctually and systematically comprised. When the perceived risk is altered through psychological, social, and cultural processes there is ultimately an effect on behavior change.

**Criteria for Selection of Articles:** A literature review including 15 articles were found through two separate searches on three databases- Pubmed, Embase, and CINAHL. Because the initial search yielded only articles related to risk perception of developing diabetes in the general population, a second search was performed to explore the risk perception of diabetes specific to VnA. Inclusion criteria from the initial search included: (1) setting in the U.S., (2) adults with prediabetes or impaired glucose tolerance, and (3) risk perception of developing diabetes. Some exclusion criteria were presentation abstracts, editorials, position statements, and literature reviews without mention of the methodology used or databases accessed. Inclusion criteria from the second search retained literature related to: (1) diabetes and (2) VnA. Excluded were any articles not based in the U. S., only looking at prevalence or BMI, and those comparing VnA to specific ethnic groups other than NHW or the general population.

**Appraisal and Synthesis of Evidence/Results:** The main themes that emerged from this literature review include: lacking perception of prediabetes being a risk for developing diabetes among Asian Americans, a link between family history of diabetes and a higher risk perception of the risk of developing diabetes, and potential misconceptions of VnA about causes and severity of diabetes.

**Implications for Nursing:** The findings from this literature review indicate the paucity of literature specific to this population and the need to further explore the risk perception of developing diabetes in VnA. Exploring risk perception will allow culturally-tailored interventions to prevent disease in this population.

**Abstract 20**

Nurse led Quality Improvement Project – Our Success Story

**Baby John, BSN, RN-BC, PCCN^1^, Jansy Sebastian, MSN, RN-BC, PCCN, CNL**

^1^Durham Veterans Affairs Medical Center, Durham, NC, USA

**Background:** About 200,000 cardiac arrests are treated in U.S. hospitals annually. It is one of the leading causes of deaths in US. Survival rates after cardiac arrest due to Ventricular Fibrillation vary in both outpatient and inpatient settings. Nurses lead and take initiatives in client centred quality improvement projects due to their unique role in the health care system. Advance education; demand for innovative, cost effective, quality work and high client satisfaction encourages nurses to take up quality improvement project. Even though all health care workers are CPR certified, and are aware of “act fast, no time to waste, every second counts in resuscitation”, panic and fear sets in causing delay in initiating quality CPR.

This quality improvement project was initiated after a code event, where the radiology technologist who witnessed the arrest was too panicked and fearful to initiate CPR. Simulation and hands on training sessions were held for all health care personnel in the Radiology department in collaboration with Simulation coordinator, Nurse manager, and radiology supervisors.

**Purpose:** The purpose of this project was to improve the confidence in providing resuscitation services at the time of emergency by using simulation of actual scenarios at bedside.

**Method:** Pre and post surveys were used to analyse the confidence level to manage the emergency with hands on training sessions using simulation of real life scenarios.

**Results:** This quality improvement project involved training sessions at bedside for the health care personnel using simulation. The staff felt more comfortable and confident in providing resuscitation and managing the emergency until the code team arrived. The analysis of pre and post survey indicates improvement in time management; knowledge and skills of the staff were improved.

**Conclusion:** This nurse led quality improvement project was successful and improved confidence to the health care personnel who participated in this project.

**Implications for Nursing:** It is important for all health care personnel to be competent to provide quality CPR until code team arrives. Frequent training and practice on high quality, timely CPR in code situations could save lives as well as improve the quality of life of the patient after successful resuscitation.

**Abstract 21**

Subethnic Differences in Background Characteristics and Survivorship Experience among Asian American Breast Cancer Survivors: Technology-based Information and Coaching/Support Program

**Chi-Young Lee, MSN, RN^1^, Sangmi Kim, PhD, MPH^1^, Eun-Ok Im, PhD, MPH, RN, CNS, FAAN^1^**

^1^School of Nursing, Duke University, Durham, USA

**Purpose:** The purpose of this study was to explore sub-ethnic variations in the characteristics of Asian American breast cancer survivors (AABCS) and examine the influences of sub-ethnicity on the women’s pain management, symptom management, perceived health status and quality of life.

**Methods:** This study was part of a larger study on Asian American breast cancer survivors’ survivorship experience and used only the data from 94 women. Questions on background characteristics and disease characteristics, the Perceived Isolation Scale (PIS), the Personal Resource Questionnaire (PRQ-2000), the Memorial Symptom Assessment Scale-Short Form (MSAS-SF), and the Functional Assessment of Cancer Therapy-Breast Cancer (FACT-B) were used to collect the data. The data were analyzed using chi-square tests, ANOVA, and hierarchical logistic and multiple regression analyses.

**Results:** There were statistically significant sub-ethnic differences in the level of education, religion, the level of acculturation, and the length of stay in the U. S. There were significant associations of sub-ethnicity to breast cancer stage (*p* = .035; Fisher’s exact test) and pain management (*p* = .000). Being Japanese was a significant predictor of pain management (*p* = .018), symptom management (*p* = .018), and quality of life (*p* = .049) after controlling for the influence of the other explanatory variables in the model.

**Conclusions:** Significant subethnic variations were found for several background features among AABCS. In addition, cancer management and health status in AABCS were influenced by country-specific cultural and demographic factors and encompasses concepts with different cultural origins.

**Implications for Nursing:** Care providers should be recognizant of subethnic diversities when providing care to AABCS. In addition, interventions for better health outcomes or management should be differentiated by sub-ethnicity and consistent with cultural context to optimize their survivorship experience.

**Abstract 22**

Trajectories Typologies of Worry in Mothers of Children Undergoing Hematopoietic Stem Cell Transplantation: A Longitudinal Study

**Eunji Cho, MSN, RN^1^, Qing Yang, PhD^1^, Sharron L. Docherty, PhD, PNP-BC, FAAN^1^**

^1^School of Nursing, Duke University, Durham, NC, USA

**Purpose:** Children undergoing high-tech intensive treatments for life-threatening conditions such as hematopoietic stem cell transplantation (HSCT) rely on parental caregiving, especially from mothers. During this treatment, mothers often experience high caregiving burden such as maternal worry which is associated with negative psychological responses. The purpose of this study was to explore trajectory typologies of worry in mothers of children undergoing HSCT and describe typology characteristics.

**Method:** Data was from a larger longitudinal, repeated measure study of maternal caregiving of children undergoing HSCT. Data was collected at 7 time-points over 1-year caregiving trajectory and we used the data from 62 mothers who completed a minimum of three data collection time points. Child Health Worry Scale was applied to measure maternal worry of child health. To identify typologies of maternal worry, latent class growth analysis (LCGA) was used. T-test and Chi-square test were conducted to compare the characteristics of across typologies. SAS 9.4 PROC TRAJ function and Mplus 7.4 were used for this analysis.

**Findings:** Maternal worry patterns in 62 mothers of children undergoing HSCT were classified into two groups: (1) low worry group (*n* = 28) and (2) high worry group (*n* = 34). Mother’s race/ethnicity, education level of the mother, number of children, and initial negative perception of the event at the beginning of child’s HSCT, and child’s diagnosis type were significantly different between two typologies.

**Conclusion and Implications for Nursing:** Nursing care should be planned with consideration of the mother’s individual characteristics and the child’s diagnosis type. Also, early assessment and early intervention should be considered to minimize the maternal worry during child’s HSCT and ultimately, to reduce the psychological caregiving burden.

**Abstract 23**

The Epigenetics of Childhood Abuse: Higher Global Methylation Levels in Whole Blood and its Associations with Childhood Abuse

**Hyunhwa Lee, PhD, MSN, APRN, PMHNP-BC^1^, Jonica Estrada^2^, Katrina Isla, BSN^1^, Sijung Yun, PhD^3^, Nada Lukkahatai^4^, Leorey Saligan^5^, Brian Walitt^5^**

^1^School of Nursing, University of Nevada, Las Vegas (UNLV), USA

^2^School of Life Sciences, UNLV, USA

^3^Yotta Biomed, LLC., USA

^4^School of Nursing, Johns Hopkins University, USA

^5^National Institute of Nursing Research, National Institutes of Health, USA

**Objectives:** Epigenetics, such as DNA methylation, has been hypothesized to explain the association between childhood adversity and health problems. This study examined how global DNA methylation patterns in blood may vary by different types of childhood abuse experience among individuals with fibromyalgia syndrome (FMS). Genome-wide DNA methylation markers were also examined using Methylated DNA Immuno-Precipitation sequencing (MeDIP-Seq) in HiSeq-2500 system.

**Methods:** Data for this study was from a fibromyalgia natural history study approved by the MedStar Health IRB, where FMS was diagnosed using the 1990 or the 2010 American College of Rheumatology criteria and Widespread Pain Index. Pain, fatigue, anxiety, depression, and cognitive function were also assessed and a detailed abuse history was obtained, including age at the time and type of abuse in the parent study. In this study, 118 FMS females’ blood samples were used for DNA purification (by QIAamp midi kit) and global DNA methylation assay (by EpiGentek MethylflashTM kit). A total of 6 FMS African American females (AAF) DNA samples were selected for MeDIP-Seq.

**Results:** A total of 52 participants were selected for analysis based on available abuse history data. Age ranged from 37 to 87 years (mean = 52.6, SD = 10.67). Approximately one third reported to experience a physical abuse; another third reported emotional abuse and one fifth reported rape. Our initial findings were not statistically significant; however when dividing the sample by age and race, abused participants have higher methylation levels compared to the non-abused group among those older than 50 (*p* < .05). Non-significant but a trend found among non-White females: abused participants with higher global methylation levels, compared to non-abused participants. From the genome-wide methylation marker data from 4 abused FMS AAFs and 2 non-abused FMS AAFs with no different clinical variables, we identified death-associated protein 3 (DAP3) hypermethylated in abused FMS AAFs.

**Conclusions:** These findings validated blood methylation as an epigenetic marker to determine the potential differences of gene expression in females with FMS and abuse history.

**Implications for Nursing:** Findings from this study could contribute to utilizing blood biomarkers for assessing and better understanding FMS symptom profiles.

**Abstract 24**

Nurses and Night shift Work: Strategies to Minimize the Effects of Night Shift Work and Sleep Deprivation on Nurses’ Health

**Letha M. Joseph, MSN, DNP, APRN, AGPCNP-BC^1^**

^1^Durham Veterans Affairs Medical Center, Durham, NC, USA

**Introduction:** Nurses opt to work night shift for several reasons. In addition to workplace requirement, factors such as convenience, family needs and extra income related to shift differentials may force nurses to work night shift. In the United States, 25% of the nursing positions require shift work.

**Description of Concept:** Night shift work has negative impacts on nurses’ physical, mental and social health. Not sleeping over a twenty four hour period or sleeping four or five hours a night during the week induces an impairment equivalent to a blood alcohol level of 0.01%. Working with impaired sleep may affect patient and staff safety and quality of care. Evidence supports the association between night shift work and several medical conditions such as certain malignancies, cardio vascular disease, gastrointestinal disease, metabolic syndrome, diabetes mellitus, reproductive disorders, sleep disorders and fatigue. Additionally, disturbed sleep - wake cycle has negative impact on mental health as evidenced by an increase in incidence of depression, anxiety disorders, and problems with concentration and memory among nurses who work night shift. Night shift work may negatively impact professional growth due to decreased opportunities for professional development. Similar to professional isolation, these nurses may also experience social isolation as they may be lacking opportunities to attend social functions and events.

There are several interventions to improve sleep hygiene that may improve the circadian rhythm and reduce health risk. American Academy of Sleep Medicine recommends sleep - hygiene, timed light exposure and planned napping as potential interventions to treat shift work disorder. There are several modifiable risk factors that are additional contributing factors to the chronic conditions mentioned above. Health promotion activities to reduce the cumulative effect of those additional risk factors may reduce the tendency of developing such conditions.

**Implications for Nursing:** It is important to create awareness among nurses on the health risks related to night shift work. Work place policy revisions and provisions to minimize the effect of night shift work on nurses’ health are beneficial. At the same time, nurses need to focus on health promotion activities. They can identify individual strategies to improve their quality of sleep, social interaction, health status and work-life balance. Nurses may get involved in professional activities to keep their career on track such as projects, national certifications and involvement in work place committees and professional organizations. Further research to identify measures to improve nurses’ health and quality of life is warranted as healthy nurses can improve health care outcomes.

**Abstract 25**

Improving Caregiver’s Knowledge of Available Caregiver Support Resources within The Veterans Affairs (VA) Medical Center and the Community

**Ladonna C. Thomas, DNP, ANP-C, VHA-CM^1^**

^1^Durham Veteran Affairs Health Care System, Durham, NC, USA

**Purpose:** The purpose of this evidence-based practice project was to develop, implement and evaluate the impact an educational intervention/Caregiver Resource Manual had with improving caregiver’s knowledge regarding resources available at the Veterans Affairs (VA) Medical Center and within the community. An additional purpose of this project was to assist the caregiver in deciding which caregiver resources located in the manual were the “right fit” for the caregiver and the Veteran for whom they were caring.

**Method:** During the Veteran’s clinic appointment, the caregiver was provided a letter with implied consent, pre- and post-questionnaire forms and a comprehensive Caregiver Resource Manual titled *Caregiver Resource Decision Manual- Putting it all Together: The One Stop Shop to Caregiver Support Resources at the VA Medical Center and in the Community*. The participants completed the pre- questionnaire form, returned it the day of the Veteran’s visit, prior to reading the manual. After reading the manual, the caregiver completed the post questionnaire form that assessed their knowledge post intervention. The questionnaires were the tools used to assess the effectiveness of the manual. These tools consisted of three questions on the pre- questionnaire and three questions on the post questionnaire that were directly geared at measuring the caregiver’s knowledge. The responses were measured with the use of a five-point Likert Scale.

**Results:** There were 15 participants; the majority were females, all wives. Both outcomes were met. The post questionnaire outcomes were considered an improvement based on caregiver’s knowledge of support resources as well as if the information in the manual decreased their stress level and gave them “peace of mind.” There was an increase from the pre- intervention scores (*M* = 66) to post intervention scores (*M* = 82). Percent increase in caregiver’s knowledge was 24%.

**Conclusion:** The greatest impact of this EBP project was that the caregivers gained a sense of knowledge and empowerment regarding available resources within the VA and the community. Providing the caregiver with a peace of mind and improving the overall health and well-being of the caregiver and Veteran was essential. Results signified empirical verification that the educational intervention was effective.

**Abstract 26**

Understanding Obesity and Cardiovascular Risks among Hmong Americans

**May Ying Ly, MSW, Ph.D^1^, Katherine Kim, Ph.D., M.P.H., M.B.A, Susan Stewart, Ph.D.**

^1^University of California at Davis, Davis, CA, USA

**Background:** Cardiovascular disease (CVD) is the second leading cause of death among Asians; however, there is limited data on cardiovascular disease for Asian-American subgroups. Risk factors to CVD include obesity. Over the last 30 years, the increasing trend of obesity has led the American Medical Association to declare obesity as a disease. The Body Mass Index (BMI) has been a useful tool to measure body fatness with categories defined as normal weight (≤ 24.9 kg/m^2^), overweight (25 kg/m^2^ to 29.9 kg/m^2^), and obese (≥ 30 kg/m^2^). Evidence is emerging that there is increased risk of diabetes and cardiovascular risk in Asian populations below the recommended cut-points for overweight and obese. For Hmong-Americans, there are no longitudinal studies on obesity and cardiovascular disease. The purpose of this study is to understand obesity and cardiovascular risks among Hmong-Americans.

**Method:** Data were extracted from electronic health records of Hmong identified patients at a local hospital ages 30-74 years old who met the study inclusion criteria for the period between 2010-2015. A total record of 173 were analyze. The World Health Organization’s standard and Asian BMI cut-points were used to determine the prevalence of obesity. The Framingham cardiovascular risk algorithm was used to calculate cardiovascular disease risk scores. Multiple regression analyses were used to determine the association between BMI and CVD risk scores.

**Results:** Using the standard BMI cut points, Hmong-Americans have an increased obesity rate comparable to non-Hispanic Whites. When compared to other Asians, the Hmong obesity rate was twice as high (32.4%) than other Asians (15.6%). Similarly with the Asian BMI cut points, the Hmong-American’s obesity rate remains higher than other Asians (51.7% vs. 29.5%). With race and gender, there was a significant association BMI and CVD risks.

**Conclusion:** The findings from this study indicated that Hmong-Americans are at an increased risk of CVD when compared to other Asian-Americans. Implications for nursing include disaggregating Asian subgroups to focus on immigrant and refugee groups such as the Hmong who have a different culture, dietary habits and migratory history from other Asians and are at increased risk of CVD.

**Abstract 27**

3D’s in 3C’s

**Sumana Gaddam, BSN, RN, CCRN^1^**

^1^Duke Children’s at DUHS, Durham, NC, USA

**Introduction:** Duke’s mission, vision and values statements are the fundamental building blocks for its success as a leading national academic health center committed to advancing health together as they convey the purpose, direction, drivers and character of our hospital. Our Vision is to discover, develop and deliver (**DDD**) a healthier tomorrow. At Duke, we strive to Deliver tomorrow’s health care today, accelerate discovery and its translation, create education that is transforming, build healthy communities, and connect with the world to improve health globally.

**Background:** Children with medical complexity represent significant pediatric population and parents/caregivers struggle to know which providers are accountable for organizing and directing their child’s overall care needs thereby shouldering the burden for coordinating these complex care needs, which leads to fragmented care and a dissatisfying experience.

**Intervention:** Duke launched its Children’s Complex Care (**CCC**) services in August 2014 and now has **10** multidisciplinary clinics where patient needs are met in one location in one day. Our primary aim is take clinical responsibility for the comprehensive, longitudinal care coordination needs of our patients. These clinics are held routinely on specific days of the month. Nurse clinicians coordinate the care of these complex patients including numerous ancillary tests, appointments and other services. Nurse experts facilitate these clinics to provide the specialized care these patients deserve.

**Outcome:**

- We found early improvements in healthcare utilization.
- By cultivating trusting relationships with our families, we gained the opportunity to provide long-term support with extreme levels of medical complexity, technology dependence and hospital lengths of stay.
- Shared medical decision-making focused on patient-centered goals, provided more satisfying experience for patients and families.
- This approach has been very successful and there is now a new inpatient multidisciplinary team called “Worley Team” named after Dr. Gordon Worley.

**Conclusion:** Every day, we continue to Discover, Develop and Deliver (**3 D’s**) improved care; putting our patients at the center of everything we do in Children’s Complex Care (**3C’s**) services at DUHS.

**Implications for Nursing:** (a) Adoption of a similar successful intervention program (b) Confidence & competence in delivering particular services (c) Explore the function of each clinic by efficacy of caregivers (d) Explore the experience of patients and families.

**Abstract 28**

Culturally Competent Coaching Strategies in Chinese-American Breast Cancer Survivors

**Shu Xu, MSN, RN, CMSRN^1^, Yun Hu, PhD, RN^1^, Sangmi Kim, PhD, MPH, RN^1^, Wonshik Chee, PhD^1^, Eun-Ok Im, PhD, MPH, RN, CNS, FAAN^1^**

^1^School of Nursing, Duke University, Durham, NC, USA

**Purpose or Objective:** Chinese-American breast cancer survivors have suffered many difficulties due to their lower healthcare literacy, English language barriers, low income, and culture challenges. Despite an increasing number of technology-based support programs for breast cancer survivors, very little is still known about the programs, especially those for Chinese-American breast cancer survivors. This presentation discusses culturally competent coaching strategies for Chinese-American breast cancer survivors that were used in a national intervention study utilizing a technology-based support program.

**Methods:** The parent study is a randomized clinical trial using a repeated measures among 330 Asian American breast cancer survivors. For this presentation, only the data from 22 Chinese-American breast cancer survivors were used. All the participants received one-on-one coaching by research nurses via weekly phone calls for three months. Research nurses were bilingual, Chinese and English, along with rich nursing experiences and understanding of Chinese philosophy and culture. The coaching topics included physical symptoms, psychosocial distresses, and ethnic cultural issues. The coaching/support strategies were based on the Bandura’s self-efficacy theory. The data were collected at three time points (pre-test, post-1 months, and post-3 months). To describe background characteristics of the participants, the data from participants’ pre-test surveys (using multiple instruments) were analyzed using descriptive statistics. To analyze the strategies used in the coaching/support, the research diaries that were written by the interventionists were analyzed using a content analysis.

**Results:** The average age was 55.5 years (SD = 9.30, range = 35 to 74 years). Of the 22 participants, 20 were immigrants from China (the mean length of stay in the U.S. = 12.9 years). Two were born in the U.S., but with Chinese heritage. Culturally tailored coaching/support strategies included: (a) adoption of Chinese traditional medicine for management of pain and insomnia; (b) application of Confucian principles to deal with psychosocial distresses; (c) using the participants’ preferred language (Mandarin or English); (d) building a trustable relationship through multiple strategies; and (e) using nursing knowledge and experience to solve daily survivorship issues.

**Conclusions:** One-on-one coaching/support using culturally tailored multiple strategies benefits Chinese-American breast cancer survivors to easily understand educational contents and to help meet their physical and psychosocial needs.

**Implications for Nursing:** Enhancing nurses’ knowledge about using culturally competent coaching strategies to provide the greater support for Asian American breast cancer survivors.

**Abstract 29**

Effects of Journal Writing on Stress and Mental Health for Mothers of Adult Children with Substance Abuse: A Pilot Study

**Yeoun Soo Kim-Godwin, PhD, RN^1^**

^1^School of Nursing, University of North Carolina Wilmington, Wilmington, NC, USA

**Purpose:** The purpose of study was to evaluate the effectiveness of the six-week’s positive writing interventions on stress and mental health in mothers of adult children with substance abuse.

**Methods:** A quasi-experimental, one-group, pretest-posttest design was used. Participants were given a journal along with the weekly writing instructions and were asked to make entries at least 3 times per week for 6 weeks. The questionnaire to measure the perceived levels of stress (PSS, Perceived Stress Scale) and mental health (Patient Health Questionnaire of PHQ-9, GAD-7, and PHQ-15 Scales) was administered both pre and post tests. In addition, saliva samples were collected at 3 different time points. The participants’ beliefs about writing journal intervention were assessed using the Credibility/Expectancy Questionnaire (CEQ) during the post-test visit.

**Results:** This study analyzed the snow balling sample of 17 mothers. Their ages ranged from 44 to 69 years, with a mean of 54.75. The baseline data indicated they experienced moderate levels of stress (*M* = 15.47 [PSS]) and rated their mental health as fairly good (*M* = 4.57 [PHQ-9]; *M* = 3.43 [GAD-7]; & *M* = 8 [PHQ-15]. A statistically significant difference was noted between pre- and post-writing intervention in stress scores (*p* = .03), but not mental health scores. The finding of repeated measures ANOVA indicated that there were no significant changes in the cortisol level (*F*(1.1267) = .805, *p* < 0.41). However, participants rated highly their writing practices on the six CEQ items: (1) logic of writing practice (*M* = 8.19); (2) confidence in managing stress (*M* = 7.88); (3) confidence recommending to a friend (*M* = 8.25); (4) willingness to perform the writing; (*M* = 7.31) and (6) belief of writing in helping with other difficulties (*M* = 7.13), using a 9 scale (1 = not at all to 9 = a great deal).

**Conclusion:** Although there was no clear conclusion in regard to the effectiveness of writing intervention practice, this current research provides some evidence to support the notion that writing interventions may be beneficial in managing stress among this population. Considering that limited resources are available for supporting family members in communities, nurses could recommend writing interventions for their clients as a self-help tool for their emotional well-being.

